# Cortical hemodynamic mapping of subthalamic nucleus deep brain stimulation in Parkinsonian patients, using high-density functional near-infrared spectroscopy

**DOI:** 10.1371/journal.pone.0245188

**Published:** 2021-01-25

**Authors:** Mahdi Mahmoudzadeh, Fabrice Wallois, Mélissa Tir, Pierre Krystkowiak, Michel Lefranc

**Affiliations:** 1 INSERM U1105, CHU Amiens-Picardie, Amiens, France; 2 Neurosurgery Department, CHU Amiens-Picardie, Amiens, France; 3 Neurology Department, CHU Amiens-Picardie, Amiens, France; 4 Laboratory of Functional Neurosciences, University of Picardie Jules Verne, Amiens, France; Preeminent Medical Phonics Education & Research Center, Hamamatsu University School of Medicine, JAPAN

## Abstract

Subthalamic nucleus deep brain stimulation (STN-DBS) is an effective treatment for idiopathic Parkinson’s disease. Despite recent progress, the mechanisms responsible for the technique’s effectiveness have yet to be fully elucidated. The purpose of the present study was to gain new insights into the interactions between STN-DBS and cortical network activity. We therefore combined high-resolution functional near-infrared spectroscopy with low-resolution electroencephalography in seven Parkinsonian patients on STN-DBS, and measured cortical haemodynamic changes at rest and during hand movement in the presence and absence of stimulation (the ON-stim and OFF-stim conditions, respectively) in the off-drug condition. The relative changes in oxyhaemoglobin [HbO], deoxyhaemoglobin [HbR], and total haemoglobin [HbT] levels were analyzed continuously. At rest, the [HbO], [HbR], and [HbT] over the bilateral sensorimotor (SM), premotor (PM) and dorsolateral prefrontal (DLPF) cortices decreased steadily throughout the duration of stimulation, relative to the OFF-stim condition. During hand movement in the OFF-stim condition, [HbO] increased and [HbR] decreased concomitantly over the contralateral SM cortex (as a result of neurovascular coupling), and [HbO], [HbR], and [HbT] increased concomitantly in the dorsolateral prefrontal cortex (DLPFC)—suggesting an increase in blood volume in this brain area. During hand movement with STN-DBS, the increase in [HbO] was over the contralateral SM and PM cortices was significantly lower than in the OFF-stim condition, as was the decrease in [HbO] and [HbT] in the DLPFC. Our results indicate that STN-DBS is associated with a reduction in blood volume over the SM, PM and DLPF cortices, regardless of whether or not the patient is performing a task. This particular effect on cortical networks might explain not only STN-DBS’s clinical effectiveness but also some of the associated adverse effects.

## Introduction

Subthalamic nucleus deep brain stimulation (STN-DBS) is a well-established, effective treatment for patients with Parkinson’s disease (PD)—especially in those with severe motor fluctuations or dyskinesia [1–4]. Most treated patients experience a dramatic overall reduction in motor symptoms and an improvement in quality of life [5,6]. However, despite rigorous preoperative selection and optimized positioning of the DBS lead inside the STN, some patients fail to benefit from this technique [7–12]. It is therefore particularly important to (i) gain a better understanding of how STN-DBS interacts with cortical networks and (ii) develop new approaches for beside monitoring of STN-DBS’s direct effect on the cerebral cortex. This mechanistic knowledge would help to elucidate STN-DBS possible implication in the clinical effects observed after surgery, individually assess postoperative outcomes, and thus improve the management of stimulation-refractory patients.

Despite recent progress, the mechanisms underlying the effectiveness of STN-DBS in patients with PD have yet to be extensively elucidated. The most recent electrophysiological explorations of the neuronal basis of STN-DBS suggests that the technique alters the spontaneous synchronous activity in the corticobasal ganglia loops; indeed, both inhibition and excitation of the various neuronal populations in the STN might provide a physiological substrate for the effects of STN-DBS [13]. Other factors might include the modulation of orthodromic and/or antidromic pathways [14] and/or the suppression of abnormal patterns of synchronized firing in the STN [15]. More precisely, STN-DBS selectively suppresses the spatially and spectrally segregated abnormal resting state in STN–cortical networks [16]. This state is characterized by abnormal coupling between the STN and the temporoparietal area and the motor/PM area in the alpha and beta bands [17,18].

Although deep brain stimulation can effectively control levodopa-refractory abnormal movements in PD, the physiological mechanisms by which this is accomplished remain obscure [19,20]. Deep brain stimulation has been extensively investigated with functional imaging. Whereas there is no doubt about the reality of DBS’s clinical effects, mechanistic data concerning the technique’s haemodynamic and/or metabolic effects are scarce. Functional imaging helps us to understand the processes associated with neurostimulation at the stimulation site but also in provides an overview of what is happening in the rest of the brain. Furthermore, functional near-infrared spectroscopy (fNIRS) is a promising method for detecting distinctive cerebral haemodynamic patterns that might be linked to disease progression and the appearance of severe symptoms. However, our knowledge does not yet extend to this aspect of STN-DBS.

Extensive functional imaging studies (mainly with PET) have provided most of our current knowledge about the overall metabolic consequences of DBS on STN-cortical networks [21–24]. The main effect of STN-DBS is a reduction in cortical metabolism in motor areas, correlated with improvement of motor function [22,23,25], while several side effects, such as recognition of emotional prosody [26] and postoperative apathy [27], have been correlated with decreased metabolism in orbitofrontal and/or dorsolateral prefrontal areas.

The subthalamic nucleus is involved in a broad range of non-motor functions, including complex cognitive and behavioural functions and networks [28]. Functional imaging studies (using 18-fluorodeoxyglucose PET, single photon emission computed tomography (SPECT), and functional MRI (fMRI)) have showed that stimulation of the STN is associated with changes in brain activation patterns not only within the nucleus itself but also in large-scale cerebral networks including associative and limbic circuits: the right anterior cingulate cortex, the right ventral striatum, the right orbitofrontal cortex, the left temporal gyrus, and the left inferior frontal/insular cortex [29–33].

Furthermore, PET, SPECT and fMRI studies have revealed that STN-DBS leads to greater regional cerebral blood flow (rCBF) [34,35] and metabolic activity [36,37] in the STN and the thalamic nuclei. However, when considering the impact of DBS close to the electrode, some researchers have variously observed non-significant increases in rCBF [38], no change in rCBF [33], or even a fall in glucose metabolism [39,40] such as that seen after damage to the STN lesions [41]. In line with the STN’s excitatory effect on the internal globus pallidus (GPi) and STN-DBS’ modulation of the STN’s output in patients with PD (See [42]), STN-DBS diminishes glucose metabolism in the globus pallidus and the putamen [36,40]. Thalamic activation increases after being released from the inhibition due to over-activation of the GPi in patients with PD, which might lead to greater rCBF in motor territories (the presupplementary motor area), associative territories, and limbic territories (the anterior cingulate) in the frontal cortex [43].

When the STN is electrically stimulated, both rCBF [34,38,44] and glucose metabolism fall in the primary motor and/or SM regions [36,39,40]. Furthermore, the reduction in rCBF is correlated with decreasing stimulation frequency—thus indirectly demonstrating that STN modulates motor cortex function [45]. The STN-DBS-induced reduction in rCBF and glucose metabolic activity has also characterized in the cerebellum (mainly in the vermis [36,40]) and in the cerebellar hemispheres [37,39,44]. Similarly, a STN-DBS-related increment in cortical activity has been observed in the DLPFC, and enhanced glucose metabolism has been described in the mesial frontal cortex and in some regions of the parietal and temporal lobes [39,40,46].

In summary, functional imaging of STN-DBS in patients with PD has highlighted the modulation of both motor and nonmotor regions, including the primary PM cortex, SM cortex, supplementary motor area, DLPFC, insular cortex, thalamus, basal ganglia (BG), and contralateral cerebellum [47–50].

In animal experiments, STN-DBS is associated with overall increments in CBF [51] and glucose metabolism [52–55]. Imaging studies in large animal models have further demonstrated the modulatory impact of DBS on motor pathway components. Min et al. reported that STN-DBS may have modulatory effects not only on both cortical and subcortical areas of the cortico-thalamocortical circuit that facilitate motor function but also on those involved in higher-level cognitive and emotional processing [56].

However, these metabolic PET studies lacked time resolution and only reflected the long-term effects of STN-DBS on cortical networks; hence, they failed to address acute effects. Furthermore, PET is an imaging technology based on the comparison of populations, and does not allow the individual monitoring of STN-DBS’s effects on cortical networks.

Functional NIRS can be used to probe the cortical haemodynamic response to STN-DBS. It has a greater time resolution, which is only limited by the timescale of neurovascular coupling. When a sufficient number of emitters and detectors are used, the high-resolution fNIRS setup that we have developed also provides high spatial resolution (at least comparable to that of PET) at the surface of the cortex. In fact, fNIRS provides information on the relative changes in cortical oxyhaemoglobin [HbO] and deoxyhaemoglobin [HbR] in response to stimuli. Like fMRI, fNIRS is based on a blood-oxygen-level dependent (BOLD) effect [57]. In contrast to fMRI, however, fNIRS studies are not limited by the magnetic field and—most importantly—the presence of a pacemaker and/or electrodes is not an exclusion criteria. Functional NIRS is therefore suitable for analyzing and mapping the acute effects of STN-DBS on cortical surface networks with fairly high time and spatial resolutions.

Functional NIRS can be used to assess haemodynamic changes and cortical activation during various motor tasks, such as a pursuit rotor task [58], a postural control task [59], kinetic movements (i.e. multijoint upper limb motor tasks) [60], maximal exercise [61], a hand-grasping task [62,63], a hand-tapping task [64], and a finger-tapping task [65,66]. Functional NIRS studies have shown that cerebral haemodynamic responses are localized in one hemisphere in simple motor tasks (such as hand grasping and finger tapping [63,67]) and in both hemispheres in complex motor tasks (e.g. using chopsticks) [68]. Furthermore, Lee et al. [68] showed that use of the dominant hand was associated with brain activation on the contralateral side on the brain, whereas use of the non-dominant hand was associated with brain activation on both the sides—suggesting the presence of functional asymmetry for the use of dominant vs. non-dominant hands during complex motor tasks.

Functional NIRS studies of cortical activation during DBS have consistently evidenced activation of the motor cortex. In a single-channel fNIRS study of patient undergoing GPi-DBS, Sakatani et al. [69] showed that [HbO] and total haemoglobin ([HbT]) increased over the motor cortex immediately after the onset of stimulation and then gradually fell as stimulation continued. Morishita et al. [70] found an increase in prefrontal cortex activity during unilateral GPi-DBS. In a recent study, Klempir et al. [71] observed that a simple finger-tapping task activated areas associated with movement planning and execution on both sides of the brain, regardless of the presence or absence of STN-DBS. In contrast, the execution of a complex motor task was associated with concentrated activity in the central cortex in the ON-stim condition and high levels of bilateral activity in the PM cortex and the supplementary motor cortex in the OFF-stim condition.

The goal of the present study was therefore to use fNIRS to characterize and map the acute haemodynamic effects of STN-DBS on cortical networks in patients with PD performing a motor task under ON-stim and OFF-stim conditions.

## Methods

### Patients

Seven patients with PD (4 right-handed and 3 left-handed; 49 to 71 years old, mean = 58.3) underwent bilateral STN-DBS (using the model 3389 quadripolar DBS lead and the Activa-PC^®^ neuropacemaker from Medtronic, Minneapolis, MI, USA). In all cases, the indication for STN-DBS was the presence of severe levodopa-related motor complications. The study population’s baseline characteristics are summarized in Table 1. The inclusion criteria were classical and similar to those defined by the Core Assessment Program for Surgical Interventional Therapies in Parkinson’s disease (CAPSIT-PD). Written informed consent was provided by the patients and the study was approved by the ethics committee (Committee for Personal Protection Nord-Ouest II) according to the guidelines of the Declaration of Helsinki of 1975 (ref ID-RCB 2013-A01297-38).

**Table 1:** Detailed patient data and sites of active contacts in the subthalamic nucleus.

Patient	Gender	(Dominant Hand)/hand movement/onset of disease	Preoperative Off Dopa	Preoperative On Dopa	Dopa sensibility	Stimulation parameters Right (0-1-2-3) and left (8-9-10-11) contact intensity (V) frequency (Hz)–pulse width (μs)	Postoperative improvement (12 months)	On Dopa/On Stim	Off Dopa/On Stim	On Dopa/Off Stim	Off dopa/off Stim
**1**	M	(L)/L/L	24	12	50%	3: 3.2–130–60	57%	12	18	15	30
						7: 1.2–130–60					
**2**	M	(R)/R/R	36	19	47%	1: 1.5–130–60	57%	17	24	26	39
						9: 2.1–130–60					
**3**	M	(R)/R/L	23	11	52%	1: 2–130–60	71.4%	8	10	16	28
						9: 2–130–60					
**4**	M	(R)/R/L	19	11	42%	1: 1–130–60	58%	5	8	5	18
						9: 2–130–60					
**5**	M	(R)/R/R	13	4	69.3%	1: 2.2–130–60	91.7%	1	5	5	12
						10: 2.5–130–60					
**6**	M	(L)/L/L	22	4	81.8%	1: 2.9–130–60	81.8%	4	7	6	22
						9: 2.5–130–60					
**7**	F	(L)RL	32	14	56%	1: 2.2–130–60	47%	18	21	13	34
						10: 3.6–130–60					

### Clinical assessment

Before surgery, the motor disability score (Unified Parkinson’s disease rating scale (UPDRS) Part III) was assessed first while the patient was in the OFF-drug condition (as defined by the CAPSIT-PD, i.e. after at least a 12-hour interruption of antiparkinsonian medication) and then in the ON-drug condition (after the administration of a single, suprathreshold dose of levodopa). For each patient, the preoperative levodopa responsiveness had to correspond to a clinical improvement of at least 50%. At the follow-up assessment, each patient’s motor disability score was rated under four conditions: ON-stim/OFF-drug; OFF-stim/OFF-drug; OFF-stim/ON-drug; ON-stim/ON-drug. The OFF-stim condition was defined as the withdrawal of STN-DBS for at least 1 hour. All patients were followed up for at least 12 months after surgery (Table 1).

### Operating technique and lead location

The operating technique has been described elsewhere [72]. Target localization was based on dedicated 3 Tesla MRI images [73]. The entire operation was performed under general anaesthesia using a stereotactic robot (Rosa^®^, Medtech, Montpellier, France) coupled with intraoperative flat-panel CT device (O-arm^®^ Surgical Imaging System, Medtronic). Neurophysiological recordings and clinical assessments (for side effects) were performed during surgery. Electrode location was checked by fusion of the postoperative CT image with the preoperative planning dataset. The STN’s boundaries in all three planes were easily determined on the preoperative MRI, and fusion of the MRI and CT images enabled us to determine the exact positions of the electrode contacts in the STN [74,75].

### Data acquisition

Functional NIRS data were acquired under the various experimental conditions, in order to characterize and map the STN-DBS’s acute effects on cortical surface networks. In order to synchronize the fNIRS analysis with the STN-DBS, 11-channel EEG electrodes were embedded in the high-density fNIRS cap; this enabled us to precisely monitor any STN-DBS-related artefacts on the scalp.

#### The NIRS imaging system

**Fig 1:**
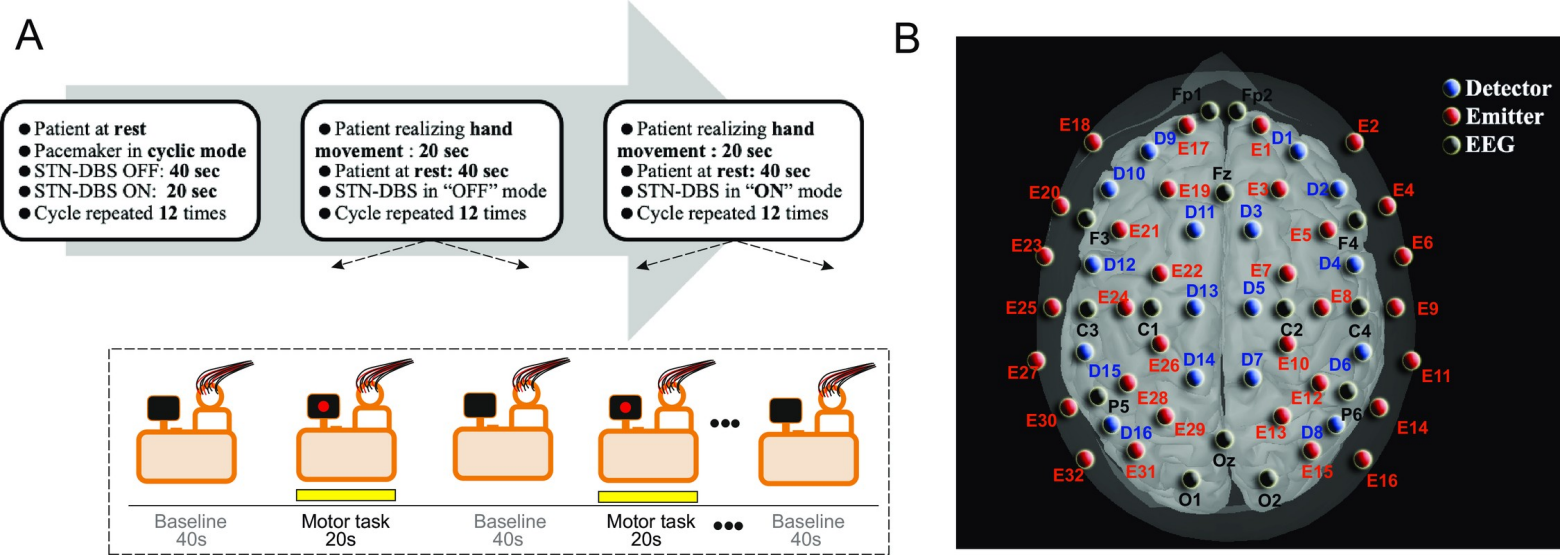
**(A)** The experimental protocol: the subject was seated on a chair 1 meter from a computer screen that displayed the task procedure. *Task 1*: the resting condition, with an alternating “ON-stim/OFF-stim” STN-DBS mode. *Task 2*: the motor task in the OFF-stim condition. *Task 3*: the motor task in the “ON-stim” mode. During each trial, the subject fixed a cross displayed on the screen for 40 seconds (resting period). As indicated on the screen, the motor task was then performed for 20 s. The motor task consisted of 10 self-paced grasping movements with repeated opening and closing of the dominant hand. This procedure was repeated 12 times. **(B)** A three-dimensional illustration of the position of the detectors and sources. In order to explore the frontal, parietal, temporal and occipital cortices with sufficiently high spatial resolution, an optical imaging cap (containing 48 optodes: 32 paired sources [690 nm, 830 nm] and 16 detectors; source-detector distance: 3 cm) was developed and used to record hemodynamic changes in the whole head.

Four multichannel, dual-wavelength (690 and 830 nm), frequency-domain fNIRS imaging systems (Imagent™, ISS, Champaign, IL, USA) were used to measure relative changes in [HbO] and [HbR] during the experimental protocol. Imagent™ is a frequency-domain tissue spectrometer in which dual-wavelength intensity-modulated laser diodes are coupled to optical fibres and gain-modulated photomultiplier tube detectors that separately record the signal at the two wavelengths. The intensity modulation frequency was 110 MHz, and the cross-correlation frequency for heterodyne detection was 5 kHz. After the reflected light had been collected in the photomultiplier tubes and demodulated, its mean intensity, modulation amplitude, and phase were determined. The lasers’ average output power was about 0.5 mW and the system’s acquisition rate was 9.1912 Hz (i.e. about one sample every 110 ms). To ensure that the spatial resolution was high enough to explore the frontal, parietal, temporal and occipital cortices, we developed a patented optical imaging cap fitted with 48 optodes (32 paired 690 nm and 830 nm sources and 16 detectors) and used it to monitor haemodynamic changes in the whole head (Fig 1).

#### Low-resolution EEG acquisition

To detect the DBS on-off timepoints in fNIRS measurements (i.e. electrical artifacts induced by DBS), continuous EEG recordings were simultaneously acquired from 14 Ag/AgCl scalp electrodes (ASA-Lab, ANT Neuro BV, Hengelo, The Netherlands) placed according to the international 10–20 system. The electrode positions are shown diagrammatically in Fig 1. Low-resolution EEG was referenced against the average of both mastoids (M1 and M2). Electrode impedances were maintained below 5 kΩ throughout the experiment. Raw EEG data were acquired at a sampling frequency of 1024 Hz and then band-pass filtered (0.53–200 Hz) with an additional notch filter (50 Hz).

### Data processing and visualization

#### Determination of the processing time window, using the EEG artifacts induced by DBS

**Fig 2:**
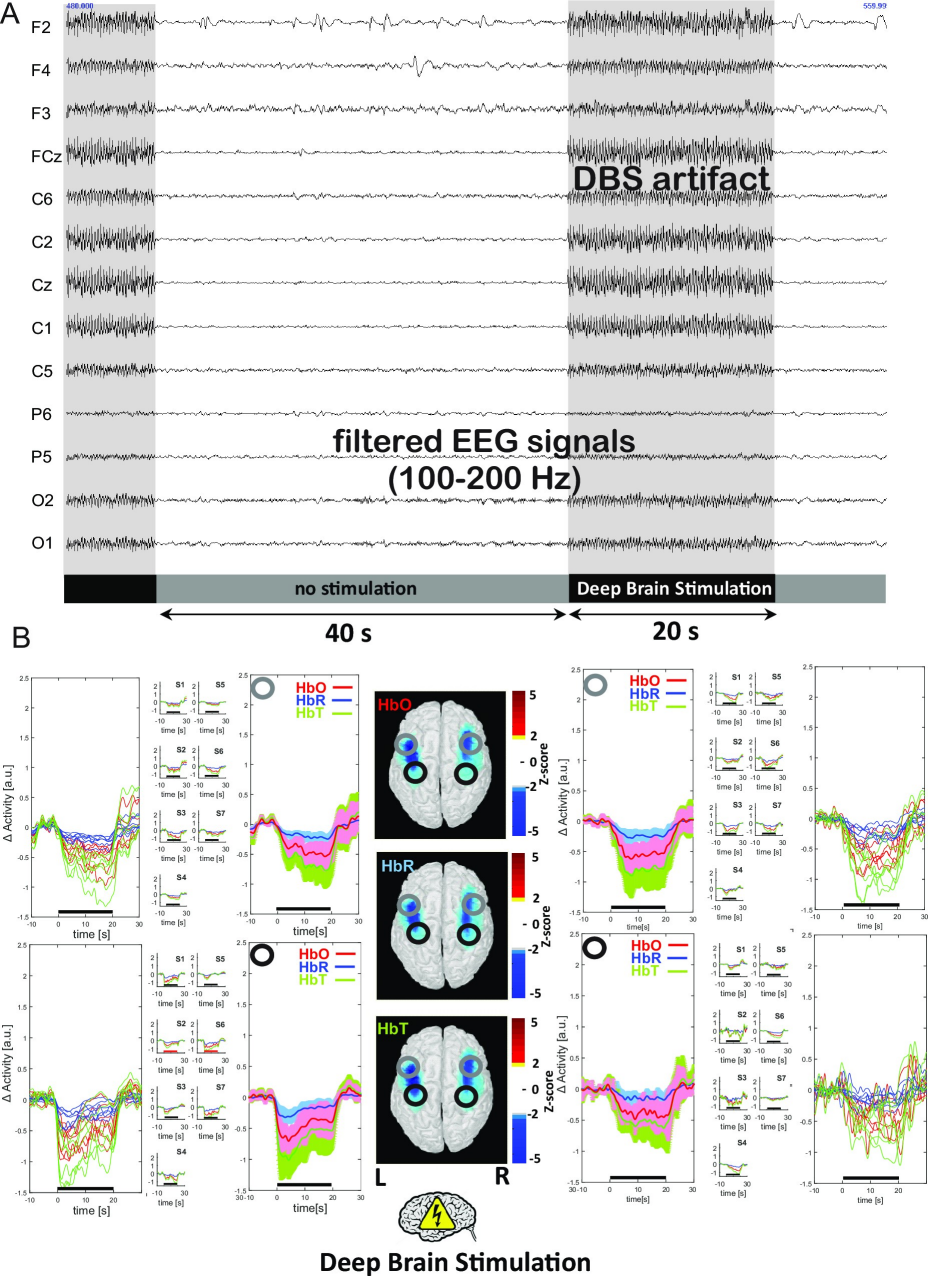
Cortical effects of STN-DBS stimulation with the patient at rest. **(A)** EEG was band-pass filtered [100–200 Hz] and brain stimulation artifacts were marked in order to determine brain stimulation periods based on optical data. **(B)** A decrease in [HbO], [HbR], and [HbT] over the bilateral SM, PM and DLPF cortical areas was observed almost immediately and was maintained throughout the duration of stimulation. The three-dimensional map of a normalized brain shows the relative variations in [HbO] during the motor task. Red indicates an increase in the relative concentration (z-score) and blue indicates a decrease in the relative concentration. The two analyzed ROIs are illustrated on each sides of the image (black: motor and premotor cortex; grey: frontal dorsolateral cortex). Individual curves are presented for all patients on one graph and each individual on a smaller graph, together with the mean ± SD of the relative changes in [HbO], [HbT], and [HbR]. The left cortex is shown on the left and the right cortex is shown on the right.

To detect the DBS periods in the fNIRS data, the EEG signal was band-pass-filtered [100–200 Hz] and DBS artifacts were marked (Fig 2). We started to monitor the haemodynamic response 10 s before the stimulation onset and stopped 10 s after the stimulation offset. The [HbR] and [HbO] signals were therefore segmented relative to the onset of each “DBS on” block. A linear detrend and a baseline correction for the [–10, 0] seconds preceding the onset of the stimulation were applied to each block. Relative changes in total haemoglobin ([HbT], i.e. [HbR] + [HbO]) were also reported.

#### Spectroscopic signal processing

The fNIRS data were analyzed offline by using a combination of in-house MATLAB scripts and a custom MATLAB fNIRS data analysis program (HomER version 4.0, available for public download and use at http://www.nmr.mgh.harvard.edu/PMI/). The first preprocessing steps were normalization of the raw intensity data from all channels and computation of a percentage change by dividing each value by the mean. To eliminate physiological noise (e.g. slow drifts and arterial pulse oscillations), intensity-normalized data were then low-pass filtered using a 3rd-order type II Chebyshev filter with a frequency cut-off of 0.5 Hz and pass-band (ripple) attenuation of 0.5 dB. The resulting delta density was used to calculate the change in concentration using the modified Beer-Lambert law, a differential path length factor of 6.0, and partial volume correction of 50. The data were then block-averaged after specifying the pre-stimulation (10 s) and post-stimulation (10 s) intervals for averaging. Block averaging was performed separately for each condition (DBS at rest, and left-hand and right-hand movements with and without DBS) for each patient individually and then for all patients.

#### Topographic map

The high spatial density afforded by our recording montage was used to generate surface-projected maps of functional brain activity. Co-registration of NIRS and MR anatomical data was performed according to the following steps. Firstly, the location of each optode (source and detector) was digitized in three dimensions with respect to three fiducial points (located on the nasion and the left and right pre-auricular points) for each individual subject, using a three-dimensional magnetic space digitizer (Fastrak®, Polhemus, Colchester, VT, USA). Secondly, standard structural MRI was used to co-register optode positions on the anatomical MR image comprising the fiducial points. Data were combined and visualized across channels by means of in-house MATLAB scripts and the optodes’ digitized locations. The script allowed us to select channels on the basis of the standard error of phase variations across blocks; this eliminates “noisy” channels for which the source-detector distance is too large to detect high levels of illumination. The in-house MATLAB scripts also enabled us to calculate z-scores over time and for the patients as a whole. After averaging in each condition (DBS at rest, and left-hand and right-hand movements with and without DBS), images of haemodynamic activations superimposed on the probe geometry were constructed using back-projection methods. For details, please refer to the HomER User's Guide at http://www.nmr.mgh.harvard.edu/PMI/.

### Statistical analysis

**Fig 3:**
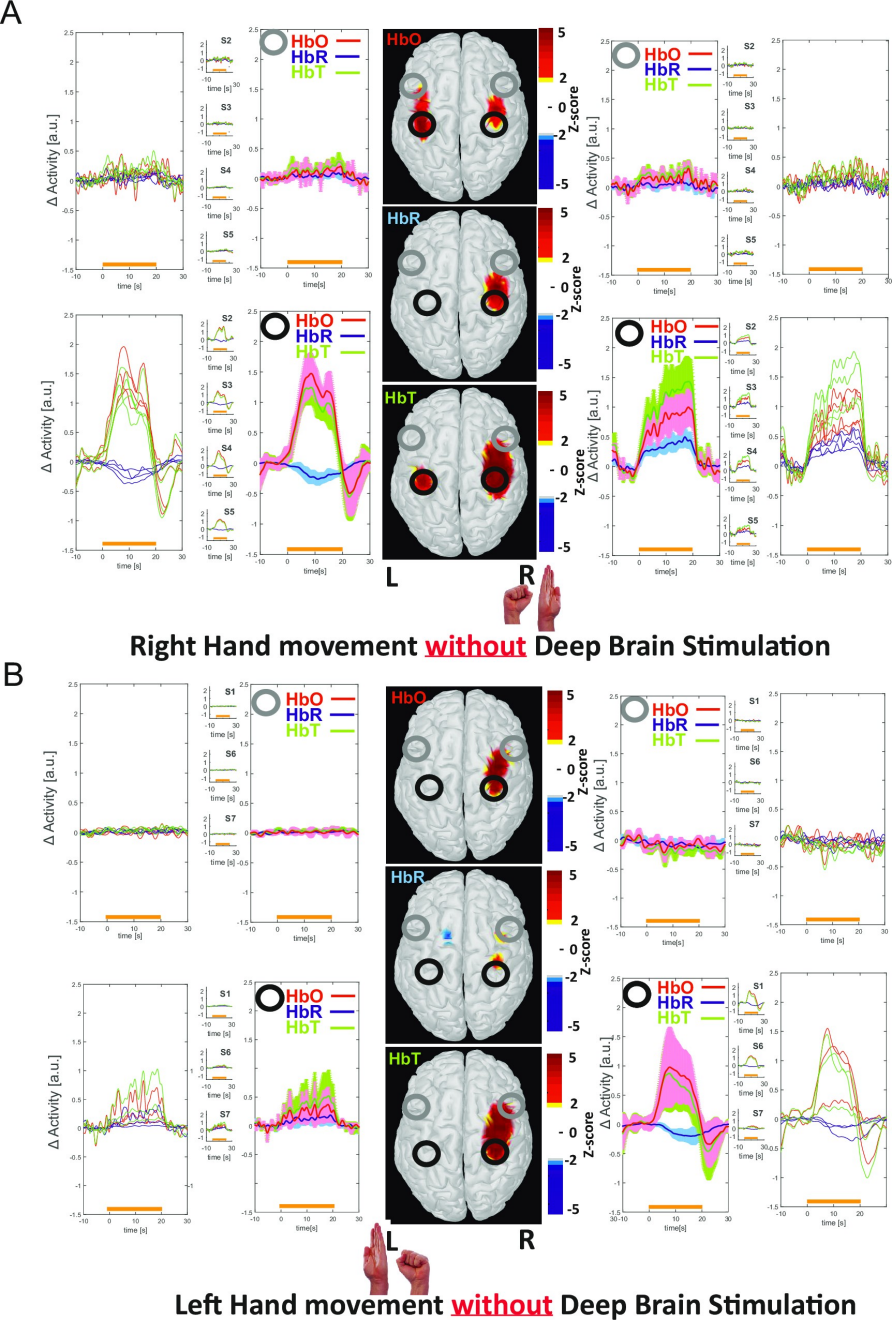
Cortical effects of STN-DBS stimulation in patients performing a motor task in the OFF-stim condition (A: right hand movement; B: left hand movement). Relative changes in cortical [HbO], [HbR], and [HbT] during hand movements in the OFF-stim condition. During movement in OFF-stim condition, an increase in [HbO] and a decrease in [HbR] (neurovascular coupling) were observed over the contralateral SM and PM areas, together with a significant but smaller change in metabolism over the DLPFC area, i.e. a concomitant increase in [HbO], [HbR], and [HbT], indicating cortical activation in the absence of neurovascular coupling.

**Fig 4:**
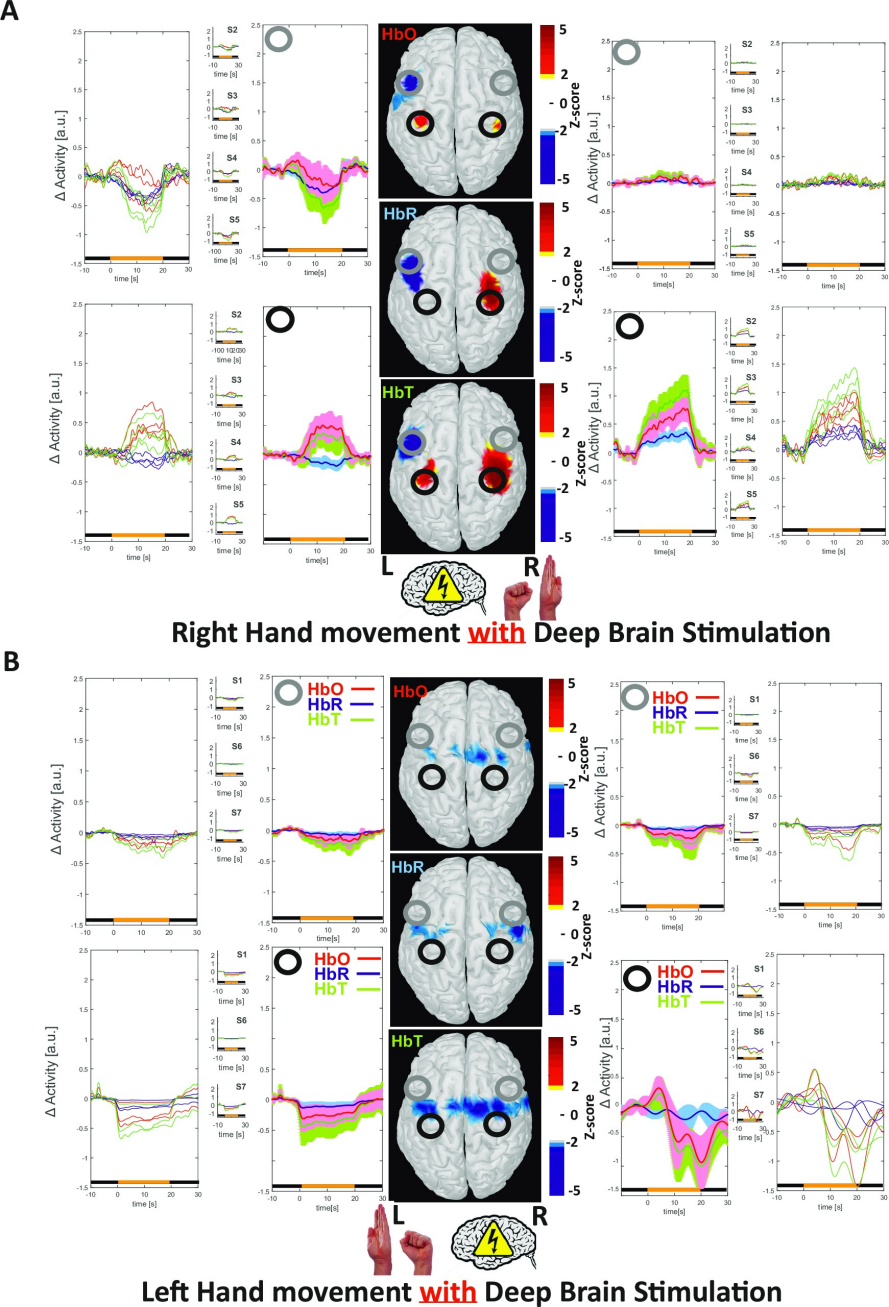
Cortical effects of STN-DBS stimulation in patients performing a motor task in the ON-stim condition (A: right hand movement; B: left hand movement). Relative changes in cortical [HbO], [HbR], and [HbT] during hand movement in the ON-stim condition. **(**A**)** In right-handed patients, concomitant cortical activation of the SM and PM cortices and deactivation of the DLPFC were observed. **(**B**)** In left-handed patients, an increase in [HbO] and [HbT] relative to baseline was observed over the right SM and PM cortices for the first 10 seconds after onset of the motor task; [HbO] and [HbT] then gradually decreased, crossing the baseline while the patient continued the motor task, and finally returned to baseline at the end of the task. This is an atypical finding: the beginning of the task resembled classical brain activation with neurovascular coupling, whereas the end of the task was associated with relative [HbO], [HbT], and [HbR] changes similar to those observed during cortical deactivation. Bilateral deactivation of the DLPFC was observed throughout the motor task.

In order to reduce multiple comparisons, we used a region of interest (ROI) approach for our statistical analysis and quantification. The ROI corresponded to the surface projections of Brodmann areas 4–6 (the motor-PM cortex) and 8–9 (the DLPFC), as defined by a higher z-score activation (z-score >2 or <-2). Only data within this ROI were analyzed. To assess the overall haemodynamic response, the area under the curve (AUC) was compared during the stimulation period (0–20 s), while reducing the number of measurements to 4 ROIs for each condition. The haemodynamic response was characterized by the amplitude and the duration. Computation of the AUC provides information on the cumulative variation of the chromophore (e.g. [HbO]) concentration over the entire stimulation period. Individual AUCs for the haemodynamic response were therefore evaluated for each ROI and each condition. Given that changes in [HbR] are often small and have a low signal-to-noise ratio (relative to changes in [HbO]), we decided to use [HbO] as the dependent variable. However, [HbR] curves are nevertheless shown in Figs 2 to 4. A paired t-test was used to test the statistical significance of the influence of STN-DBS under the various conditions. Individual AUCs for the haemodynamic response were thus evaluated for each ROI and each condition (right hand movement: df = 15 [4 subjects × 4 channels per ROI -1]; left hand movement: df = 11 [3 subjects × 4 channels per ROIs -1]). To reduce type I errors, we used Bonferroni method’s to adjust the statistical significance for each pairwise comparison. The level of statistical significance level (α) is divided by the number of ROIs (M): αBonf = α/M. In the present analysis, the threshold for statistical significance (p) was set to [0.05/4 (α_Bonf_ = 0.0125), 0.01/4 (α_Bonf_ = 0.0025) or 0.001/4 (α_Bonf_ = 0.00025)].

### Experimental procedures

The fNIRS data were acquired 12 months after surgery. Before the fNIRS acquisition, we checked that each patient had understand the task procedure and was able to perform the motor task in the OFF-stim condition. The therapeutic contacts and stimulation parameters used were those selected by the expert neurologist, based on the documented therapeutic effects. In the OFF-drug condition, all patients were evaluated by fNIRS throughout the entire experimental procedure. During the experiment, the subject was seated comfortably in a quiet, dark room. The chair was placed 1 meter from the computer screen displaying the task procedure.

#### Task 1: The resting condition, with alternating ON-stim and OFF-stim modes

We initially characterized the acute cortical effects of STN-DBS by evaluating the relative changes in [HbO] and [HbR] during alternating OFF-stim and ON-stim conditions at rest. The stimulator was therefore set to a cyclic mode (20 s on and 40 s off) and the procedure was repeated 12 times. The duration of the stimulation block (20 s On) was defined in order to increase the haemodynamic response’s signal-to-noise ratio. The time-to-peak of the haemodynamic signal in adults was about 5 to 6 seconds. In order to obtain a robust haemodynamic response, a 20-second block was therefore expected to encompass the cerebral haemodynamic response peak or even a plateau. The interval between two stimulation blocks (40 s Off) was selected to allow the haemodynamic variables to return to baseline (at least 20 s). Lastly, a cyclic mode was chosen because the independent ethics committee had specifically asked us to minimize the duration of the experimental procedure.

#### Tasks 2 and 3: The motor task in OFF-stim and ON-stim modes

To analyze the effect of STN-DBS on the cortical surface motor networks, the fNIRS data were monitored during a motor task in both OFF-stim and ON-stim modes. The two conditions were then compared with regard to the relative changes in [HbO] and in [HbR]. During each trial, the subject fixed a cross displayed on the screen for 40 seconds. This corresponded to the resting period; the patient was at rest and did not perform any movements or any cognitive activities other than watching the screen. An instruction to perform the motor task was then displayed on the screen, and the patient performed the task for 20 s. The task consisted of 10 self-paced grasping movements with repeated opening and closing of the dominant hand (right hand n = 4; left hand n = 3). This task was repeated 12 times.

## Results

### Patients and active contact locations

Six men and 1 woman (mean (range) age: 58 (49-71)) were evaluated. The mean preoperative levodopa response was 56.9%. The mean UPDRS III improvement at 12 months was 68.4% (mean (range) OFF-drug score before surgery: 24.1 (13–36); mean ON-stim/ON-drug score: 8.6 (1–17); mean ON-stim/OFF-drug score after 12 months: 13.3 (5–24)). All contacts used in this study were located within the STN, as confirmed by (i) a typical electrophysiological recording of the STN for each patient (i.e. penetration of the tip of the electrode into the STN was confirmed by a sudden increase in background activity and single-cell burst activity of spontaneously active neurons [76]), and (ii) the presence of an active contact inside the STN nucleus (after the fusion of preoperative MRI and postoperative CT images) [74,75].

### Individual fNIRS maps

#### The resting condition, with alternating ON-stim and OFF-stim modes

In all patients, and in comparison with the 40 s OFF-stim period, a 20-second period of bilateral STN-DBS stimulation significantly reduced Parkinsonian symptoms and immediately induced a concomitant, significant (p<0.05) bilateral decrease in [HbO], [HbT] and, to a lesser extent, [HbR] in the SM, PM, and dorsolateral prefrontal (DLPF) cortices (Fig 2). The trough in [HbO], [HbR], and [HbT] was observed between 5 and 20 s after the onset of stimulation. The return to baseline took 5 to 10 s and was followed by a rebound effect (i.e. an increase in [HbO] and [HbT]) that lasted for 5 to 10 s. There was no correlation between the intensity of stimulation and the magnitude of the decrease in [HbO] in the SM, PM and DLPFC areas. Individual and averaged results for the PM cortex and DLPFC are shown in Fig 2.

#### The motor task in the OFF-stim condition

**Fig 5:**
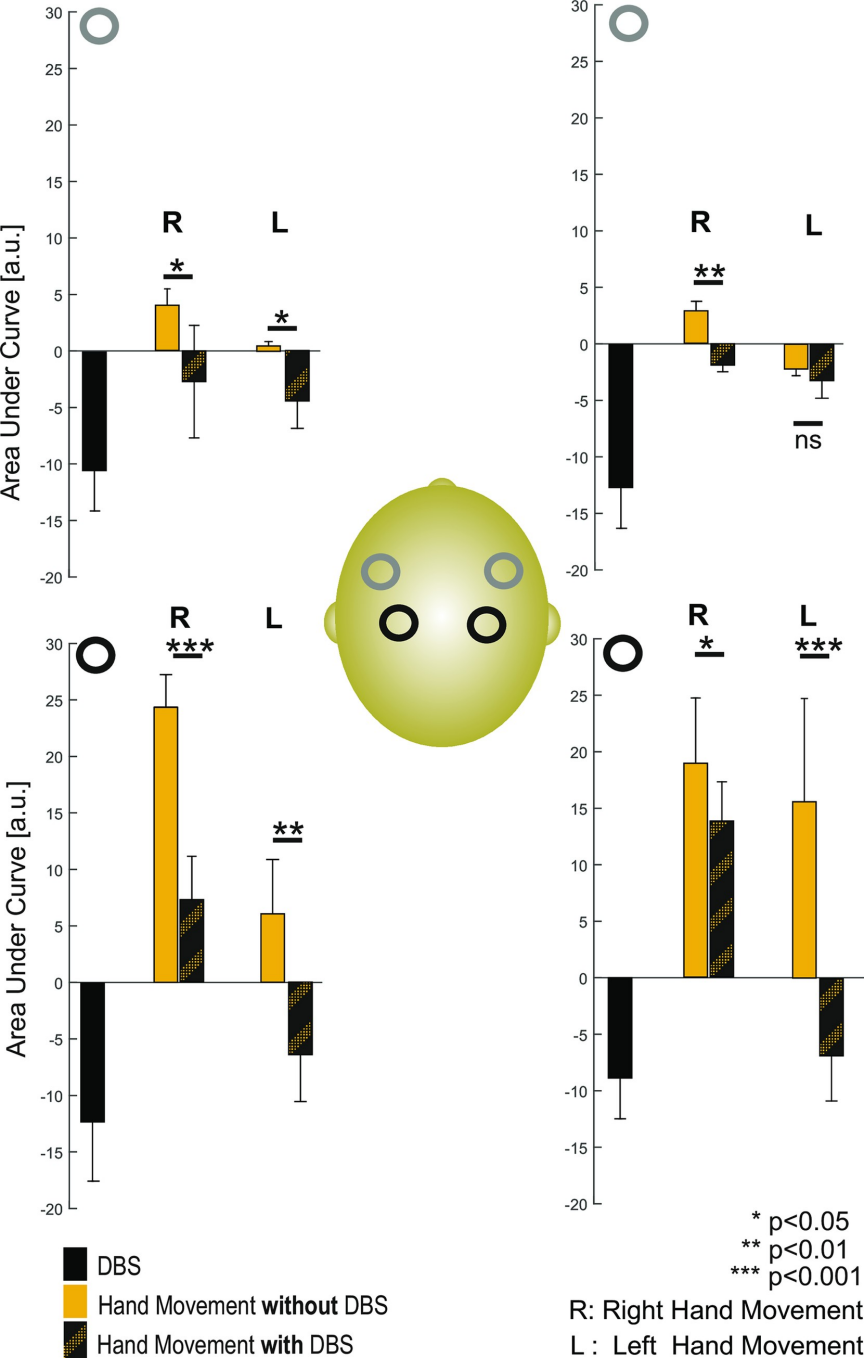
Statistical analysis of relative changes of [HbO] during the three experimental tasks in right- and left-handed patients over the right and left SM, PM and DLPF cortices. A significant decrease in [HbO] was observed over the bilateral SM/PM and DLPF cortices when the patient was at rest in the ON-stim condition. A significantly smaller increase in [HbO] was observed over the bilateral SM/PM and DLPF cortices when the patient was performing the motor task in ON-stim condition (vs. the OFF-stim condition). However, in left-handed patients, a trend towards a marked decrease in [HbO] in the right DLPFC during in the ON-stim condition (relative to the OFF-stim condition) was observed.

In the OFF-stim condition, all the patients presented severe Parkinsonian symptoms during the motor task. In all patients, neurovascular coupling was observed over the SM and PM cortices contralateral to the hand movement; this consisted of an increase in [HbO] and [HbT] for the first 10 seconds during the motor task, along with a progressive decrease in [HbR]. An increase in cerebral blood volume (CBV) was observed over the SM and PM cortices ipsilateral to the hand movement; in most patients, this consisted of an increase in [HbO], [HbR], and [HbT] throughout the stimulation period. A slight bilateral increase in [HbO] and [HbT] over the DLPFC was observed during the motor task in right-handed patients, whereas no changes in [HbO] and [HbT] in the DLPFC were observed in left-handed patients (Figs 3 and 5). The data are shown in Fig 3, and the statistical analysis of [HbO] changes is described in Fig 5.

#### The motor task in the On-stim condition, for right-handed patients

All patients presented significantly fewer Parkinsonian symptoms in the ON-stim condition than in the OFF-stim condition–meaning that the motor task could be performed more rapidly and more easily. In right-handed patients, neurovascular coupling was observed over the left SM and PM cortices during right-hand movement; this consisted of an increase in [HbO] and [HbT] and a concomitant, progressive decrease in [HbR] for the first 10 seconds of the motor task. However, the increase in [HbO] was significantly smaller (p<0.001) than that observed in the OFF-stim condition (Fig 5). In right-handed patients, an increase in CBV was also observed over the right SM and PM cortices during right-hand movement, consisting of an increase in [HbO], [HbR], and [HbT] for the first 10 seconds during the motor task. However, this increase was less pronounced (p<0.05) than that observed during the motor task performed in the OFF-stim condition (Figs 4 and 5). In right-handed patients, a decrease in CBV was observed over the DLPFC contralateral to the right-hand movement; it consisted of a decrease in [HbO], [HbR], and [HbT] for the first 10 seconds of the motor task. This response was the opposite of that observed during the motor task in the OFF-stim condition. Furthermore, the decrease in CBV (i.e. [HbO]) observed in the DLPFC was significantly more pronounced in the ON-stim condition than in the OFF-stim condition (Fig 5) (p<0.05 for the left DLPFC and p<0.01 for the right DLPFC). Given that no changes in [HbO] and [HbR] were observed in the ipsilateral cortical area, the difference between ON- and OFF-stim modes was less pronounced.

#### The motor task in the ON-stim condition, for left-handed patients

In left-handed patients, weak, nonsignificant neurovascular coupling was initially observed over the SM and PM cortices contralateral to left-hand movement; this consisted of an increase in [HbO] and [HbT] and a concomitant decrease in [HbR] for the first 5 seconds of the motor task immediately, and then reverse neurovascular coupling (characterized by a non-significant decrease in [HbO] and [HbT] and no change in [HbR]). The overall response was contrary to that observed during the motor task in the OFF-stim condition (Figs 3B vs. 4B). In left-handed patients, a slight, non-significant decrease in CBV was consistently observed over the SM and PM cortices contralateral to left-hand movement and in the left and right DLPFCs; this consisted of a decrease in [HbO], [HbR], and [HbT] during the motor task. The overall response was contrary to that observed during the motor task in the OFF-stim condition (Fig 5).

## Discussion

In line with previous studies, the ON-stim UPDRS III score one year after surgery was 68% greater than the preoperative OFF-stim score and 44.8% greater than the postoperative ON-stim score [2,6,77,78]. These differences confirmed that at least one active contact was located in the STN for each Parkinsonian patient, and so enabled us to analyze the cortical effect of STN-DBS in this homogeneous population.

### Cortical haemodynamic mapping for patients at rest in the ON-stim condition

Our results confirmed that cortical activity falls following STN-DBS, i.e. a decrease in [HbO], [HbT], and [HbR] was observed over the SM, PM, and DLPF cortices. This effect is similar to the hypometabolism described in the same cortical areas in PET studies [21,23,79–81] and might correspond to cortical deactivation [82–84]. In line with PET studies [22], this deactivation is concomitant with the changes in clinical symptoms observed in patients with PD when STN-DBS is switched on. This timing is similar to that observed in clinical practice, with the almost immediate appearance of a clinical effect when the stimulator is switching on (if the stimulation intensity threshold is exceeded) and rapid disappearance of a clinical effect when the stimulator is switched off [85,86]. It is known that STN-DBS interacts with the associative DLPFC, as well as with the SM and PM cortices. The DLPFC’s involvement in the response to STN-DBS might be linked to the anatomical targets. The somatotopic organization of the motor and associative territories of the STN is partly disrupted in Parkinsonian patients. The motor and associative territories are separated by functional gradients—indicating that neurons in the central part of the STN are connected to both the SM and associative cortices [87–90].

### Cortical haemodynamic mapping of patients performing the motor task in the OFF-stim condition

In all patients, typical neurovascular coupling (i.e. an increase in [HbO] and [HbT] and a simultaneous but less pronounced decrease in [HbR]) was observed over the contralateral SM and PM cortices immediately after the onset of the motor task. Simultaneously, significant increases in [HbO], [HbT] and, to a lesser extent, [HbR] were observed in the ipsilateral hemisphere in all patients during the motor task. This simultaneous increase in [HbO], [HbR], and [HbT] has been previously described in healthy subjects performing a motor task [91–95]. The corresponding increase in blood volume might be related to “cortical participation”, i.e. an increase in cortical haemodynamics with no associated neurovascular coupling.

In addition to this bilateral activation of the SM and PM cortices in patients with PD performing a motor task, [HbO], [HbR], and [HbT] in the DLPFC tended to increase slightly (suggesting the activation of this area). The DLPFC is not usually involved in motor tasks performed by healthy people [96]. In the present study, bilateral participation of the DLPFC was observed in right-handed patients, whereas none was observed in left-handed patients. The AUC for activation was less pronounced in the DLPFC than in the SM and PM cortices. Involvement of the DLPFC is consistent with the hypermetabolism observed in PET studies and the changes of cortical neuron excitability seen in patients with PD [22,23,80,97]; hence, the DLPFC might be involved as a cortical compensatory mechanism during the performance of a motor task.

### Cortical haemodynamic mapping in the ON-stim condition in patients performing a motor task

In patients in the ON-stim condition, the motor task induced concomitant PM and SM activation and DLPFC deactivation. Right hand movement during STN-DBS induced typical neurovascular coupling, with an increase in [HbO] and [HbT], a concomitant decrease in [HbR] in the contralateral PM and SM cortices, and a simultaneous increase in blood volume in the ipsilateral SM and PM areas.

However, the increase in [HbO] following the initiation of the motor task in the ON-stim condition was significantly smaller (p<0.001 for the contralateral increase and p<0.05 for the ipsilateral increase) than that observed during the motor task in the OFF-stim condition—in line with the deactivation associated with STN-DBS at rest [21,23,79–81] and which would minimize the increase in neurovascular coupling by decreasing the amplitude of the activated network.

Lastly, the DLPFC was deactivated when right-handed patients performed the motor task in the ON-stim condition. This haemodynamic response to the task during STN-DBS was the opposite of that observed in the OFF-stim condition.

The responses over the SM, multi PM, and DLPFC were not significant in left-handed patients with PD. Nevertheless, a biphasic response to the motor task in the ON-stim condition was consistently observed over the PM and SM cortices in each patient. Surprisingly, this response did not correspond to that observed in right-handed patients. Although the difference vs. right-handed patients was not significant, STN-DBS in left-handed patients induced ipsilateral deactivation of the SM and PM cortices and concomitant bilateral deactivation of the DLPFC. The less marked increase in [HbO] in these areas of the cortex was significant, except in the ipsilateral DLPFC in the ON-stim condition vs. the OFF-stim condition. The amplitude of the response was lower when the motor task was performed in the ON-stim condition, again suggesting that STN-DBS deactivates these networks.

Our results agree with those presented by Klempir et al. [71], suggesting that STN-DBS normalizes activation patterns in the central cortex. Klempir et al. [71] observed less widespread activation during complex gait movement in the ON-stim condition than in the OFF-stim condition. The activity was concentrated towards the central cortical areas, which resulted in an increase in task efficiency; this might have been due to STN-DBS’s ability to “normalize” brain connectivity (i.e. to produce a pattern that more closely resembles that of healthy controls) [98]. Even though we did not observe a spatial reduction in activation of the central cortex during hand movement, the relative decrease in the amplitude of the response observed over these regions might correspond to a reduction in the size of the area activated in a more complex task [71].

In the present study, we observed a few differences between right-handed and left-handed patients—notably during ON-stim hand movement. This suggests that motor control is not impacted in the same way by STN-DBS in right-handed and left-handed individuals. Furthermore, the decrease in activity in the DLPFC during hand movement in the ON condition was not observed by Klempir et al [71].

### New insights into cortical haemodynamic mapping

The present results shows that STN-DBS’s neuromodulatory output (leading to a decrease in cortical haemodynamics) did not depend on cortical activity, i.e. cortical activity was reduced significantly upon the onset of STN-DBS during hand movement in all patients. The observed decrease in cortical blood flow over the SM, PM, and DLPF cortices (even when the patient was performing a motor task) strongly suggests that STN-DBS relieves Parkinsonian symptoms by reducing abnormal cortical overactivity at rest in the SM, PM and associative systems. The presence of specific neurovascular coupling over the SM and PM cortices during hand movements in right-handed patients (while [HbO], [HbR], and [HbT] continued to decrease in the DLPFC) is particularly interesting. In our opinion, this observation could be explained by previous studies in which PD was associated with (i) noisy output from the BG and (ii) cortical activity that was hypersynchronized with the BG network [15,99–103]. The reduction in cortical metabolism is linked to suppression of excessive beta synchrony observed in patients with PD after STN-DBS [15,104]. Hence, STN-DBS might exert its effects by interfering with noisy output from the BG and by modulating cortical input [101,104,105]. Carron *et al*. showed that antidromic activation of a network is sufficient to reverse the abnormal pattern of synchronization and emphasized the potential role of cortical interneurons in cortical desynchronization [101]. By inducing antidromic spikes via the hyperdirect pathway, STN-DBS might inhibit the cortical pyramidal neurons connected to the nucleus. This inhibition might explain our observation of the ON-stim reduction in cortical blood flow at rest, the concomitant cortical activation observed over areas involved in the task, and cortical deactivation over areas not involved in the task. The decrease in [HbO], [HbR], and [HbT] over areas of the cortex connected to the STN may reflect the antidromic effect of STN-DBS. During a motor task, the involved cortical neurons generate spikes in the hyperdirect pathway; these spikes cancel STN-DBS’s antidromic action. At the same time, STN-DBS evokes orthodromic axonal spikes that are responsible for low-amplitude, postsynaptic noise and that trigger output from the BG to the cortex [102,106–108]. This mechanism might also explain our observation in left-handed patients during the motor task: an increase in [HbO] and a decrease in [HbR] over the SM cortical area (as seen during brain activation with neurovascular coupling) but a decrease in [HbO] and [HbT] at the end of the task (similar to that described during STN-DBS in resting patients). In these left-handed patients, the overall decrease in [HbO] might be linked to a direct antidromic effect on the cortical network. Treatment with STN-DBS might improve function by modifying both BG output and cortical overactivity, as these two mechanisms may allow cortical structures to more effectively compensate for the underlying deficit by inducing more specific task-related neurovascular coupling.

This mechanism might also explain the findings reported by Sakatani *et al*. and Murata *et al*. [69,109]. These researchers observed that ventrointermediate nucleus (Vim)-DBS and Gpi-DBS had different cortical impacts: Vim-DBS induced a decrease in blood flow over the SM cortex (similar to that induced by STN-DBS), whereas Gpi-DBS induced an increase in the cortical haemodynamic response. This difference might be related to the stimulated nuclei’s anatomic connections to the cortex: the STN and the Vim have a direct (hyperdirect) connection to the cortex, whereas the Gpi does not [110–112]. Moreover, Sakatani et al. [69] results suggested that the GPi stimulation might activate a possible functional connection between the GPi and the prefrontal cortex, the threshold of which is higher than those of the connections between the GPi and the motor/premotor cortex. Sakatani et al. [69] suggest further studies, such as simultaneous measurements of fNIRS and PET that may clarify the possible mechanism and also the inconsistency between the fNIRS and PET findings. In addition, Sakatini et al. also showed CBF changes during several minutes’ measurement epochs using fNIRS. While PET-CBF image acquisition is typically 2 minutes, the current study used real time monitoring (one sample every 110 ms) of cerebral hemodynamic changes with 20 and 40 seconds (ON-stim vs. OFF-stim conditions) in close temporal proximity (Fig 1B). For example, Sidtis et al. 2004 [113] demonstrated that CBF patterns associated with different tasks carried over to adjacent rest conditions separated by 8 minutes. Furthermore, the fNIRS data (relative hemodynamic activities) in the current study are not normalized for individual global effects, while in PET, the effects of DBS were essentially global. Therefore, the inconsistencies in different studies may also be due to these temporal resolution, scanning time differences and temporal effects (i.e. rest-task temporal proximity).”

**Fig 6:**
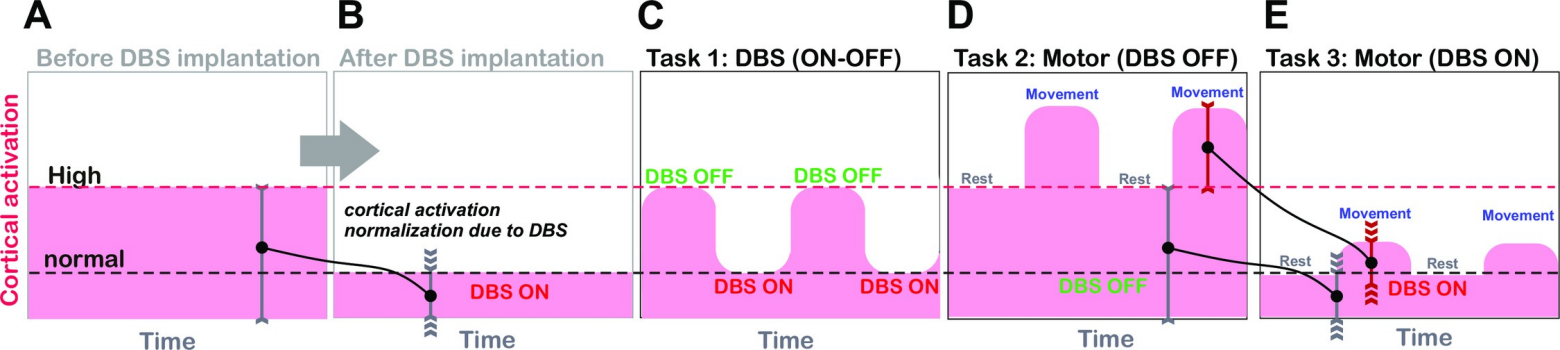
Show the level of cortical activity **(A)** before DBS implantation on parkinsonian patients **(B)** After DBS implantation, the level of cortical activity had been normalized **(C)** task 1, The resting condition, with alternating ON-stim and OFF-stim modes, during experiments with a cyclic (ON-stim/OFF-stim mode), the OFF condition shut down mechanisms that in turn decreased cortical activity and increased the brain’s metabolic activity **(D)** task 2, the motor task in OFF-stim mode, **(E)** task 3 the motor task in ON-stim mode.

Since our patients had undergone STN-DBS for about one year, the level of cortical activity had been normalized (i.e. reduced) (Fig 6A and 6B). During experiments with a cyclic (ON-stim/OFF-stim mode), the OFF condition shut down mechanisms that in turn decreased cortical activity and increased the brain’s metabolic activity (Fig 6C). Likewise, the observed relative decrease in hemodynamic activities depended on the activation state during the “baseline” period. Thus, comparisons of movement-related cortical activity (Task 2 vs. 3) must take account of differences in the “steady-state baseline” (Fig 6D and 6E). The fact that the neurovascular coupling observed during hand movement was less pronounced in Task 3 (ON-stim) than in Task 2 (OFF-stim) might be related to the level of steady-state cortical activity, which had already been normalized by the STN-DBS. These inter-task differences in the steady-state period complicate comparisons of relative changes; hence, absolute values of [HbO] and [HbR] would be required to provide an absolute cortical activation index.

Other researchers have also used a cyclic mode to study the immediate effects of acute DBS [114–116]. It seems very likely that STN-DBS exerts its effects through several mechanisms [19,20,117–119], including the interruption of neural signals (i.e. overriding pathological BG activity), interference with inhibition and excitation, the desynchronization of low-frequency oscillations, and the modulation of neurotransmitter and astrocytes. However, a detailed characterization of the neurophysiological, neurochemical and metabolic mechanisms of DBS (including the anatomic connections that mediate the disruption of information flow and the “normalization” of activity within the BG-corticothalamic network) and optimization of DBS therapy will require the use of an absolute brain activity index.

Lastly, we observed that STN-DBS induced a reduction in activity in the SM, PM and DLPF cortices, regardless of whether or not the patients were performing a motor task. This “cortical deactivation” of the DLPFC might contribute to the clinical improvement seen in PD patients on STN-DBS. In the present study, we observed that the abnormal cortical activation in the DLPFC in patients performing an OFF-stim motor task was “corrected” by the cortical deactivation of the DLPFC induced by STN-DBS. In other words, DLPFC deactivation might also be involved in the side effects of STN-DBS, such as postoperative apathy or cognitive deterioration [26,120–122]. In this context, the best candidates for STN-DBS stimulation would need to present a form of “cortical supply” in order to benefit from the effects of stimulation on motor symptoms, while simultaneously avoiding side effects due to DLPFC deactivation. It would therefore be very useful to (i) identify preoperative haemodynamic markers of these side effects [9,120] and (ii) use postoperative fNIRS mapping to monitor haemodynamic parameters and then adjust stimulation parameters appropriately. Changing the STN-DBS parameters (intensity, pulse width, etc.) might decrease the stimulation of STN neurons connected to the DLPFC. This approach could be particularly useful in patients experiencing cognitive side effects of STN-DBS surgery. Overall, individual preoperative mapping and postoperative monitoring with fNIRS provide an opportunity to decrease the cognitive side effects of STN-DBS.

The several studies have found that DBS modulates the overall neuronal network by changing the connectivity within the cortico-thalamocortical circuit, rather than simply activating or inactivating the latter [123]. Deep brain stimulation usually normalizes pathological brain activation patterns [98]. Even though stereotactic lesions and DBS have similar clinical effects, the mechanisms differ. However, the literature data are contradictory–probably due to inter-study differences in methodologies and inter-patient variability [70].

Furthermore, the inter-patient variability in activation observed following GPi-DBS [70] was not present in our STN-DBS study, unless that the amplitude of the haemodynamic responses varies with patients. Moreover, in current study, DBS lead placement in the present study was reviewed with post-operative CT and MRI [124]; we confirmed that all the contacts were well positioned in the STN.

Although many researchers have analyzed the impact of unilateral vs. bilateral neurostimulation [70] or ON-stim vs. OFF-stim conditions [71], few have compared patterns of cerebral activation under various clinical conditions (e.g. clinically effective vs. clinically ineffective DBS). Furthermore, functional imaging (SPECT and PET) is usually performed weeks or even months after the clinical situation has stabilized and the DBS parameters have been optimized. In contrast, fMRI studies must be performed soon after implantation of the intracerebral electrodes and so can only provide information on the acute effects of DBS. Functional MRI performed a few days after electrode implantation might be biased by microlesions and local oedema around the electrodes—conditions that frequently take several days to abate. Acute microlesions and oedema *per se* influence the function of motor circuits and alter clinical symptoms [125–127]. Furthermore, the type and dose of medication during the first 6 months after surgery can vary and so might also influence brain activation.

Morishita et al. [70] reported fNIRS data after one month of follow-up; the researchers concluded that an effect of microlesions could not be ruled out. Furthermore, oedema makes it more difficult to detect the BOLD signal, which is crucial for fMRI. The challenge then is to know whether the mechanism of DBS observed with fNIRS is the same as that observed with fMRI, PET or SPECT performed months after the operation on optimized patients having adapted to chronic DBS. Likewise, inconsistencies may be due to inter-patient differences in the types and severity of clinical manifestations. The motor task is another source of variability. For example, Lee et al.’s [68] fNIRS study of healthy right-handed subjects revealed a significant difference in brain activity between the dominant and non-dominant hands during a complex motor task. When the non-dominant hand was used, brain activity in both hemispheres was high. In contrast, Kashou et al. [63] did not observed a statistically significant difference in the level of activation for a finger-opposition task versus a grasping task—suggesting that the two motor tasks can be used interchangeably for the assessment of motor function with fNIRS. In addition to the resting state, DBS has been regularly studied in patients performing voluntary movements. The particular variations from the norm in each study are probably related to the features of the motor task used, whether the movements are self-generated or not, and patient’s disease stage. In most imaging investigations, the challenge is that variations in motion-related activation may not necessarily be related to DBS and might simply reflect dissimilarities in motor performance. Notwithstanding these caveats, functional imaging has made extraordinary contributions to our knowledge about DBS. In the future, we expect fNIRS, fMRI, perfusion SPECT and PET to have important roles in the exploration of the mechanisms of neurostimulation.

Functional NIRS measurements are limited to the outer cortex and have a moderate spatial resolution (around 1 cm) [128–130]. The measurement depth in fNIRS mainly depends on the source-detector distance [131–133]. The depth of the cortical surface relative to the scalp differs from one region of the cortex to another but does not exceed 2 or 3 cm. Unlike fMRI, fNIRS measurements cannot provide information about anatomic landmarks and are not suitable for examining structures such as the thalamus or for studying relationships between cortical and subcortical structures. It is important to note that the relationship between the cerebral haemodynamic signal recorded by fNIRS and the associated neuronal activity is complex. Firstly, cortical haemodynamic changes (i.e. the fNIRS signal) may reflect modifications in firing rates and subthreshold activity and so may not distinguish between areas of neuronal inhibition and excitation [134–136]. Secondly, cortical haemodynamic changes reflect the activity of many neurons and astrocytes and are thus unable to differentiate between large changes of activity in a small number of cells and small changes in a large number of cells [134–136]. Furthermore, and although microrecordings of the local field potential and multi-unit activity present a direct measure of neuronal activity, fNIRS measurements of (de)activation merely provide an indirect guide to neuronal activity based on local cerebral haemodynamic and metabolic functions [132]. In fNIRS, activation is caused by higher/lower local blood flows and/or volumes (rCBF and regional CBV) containing higher/lower oxygen concentrations [132]. Therefore, neither fNIRS nor the other cerebral haemodynamic/metabolic imaging techniques can determine which neuronal processes occur at DBS site; whatever activation or deactivation is seen in fNIRS cannot be readily equated to neuronal excitation or inhibition. Despite these issues, fNIRS can nevertheless provide an overall evaluation of neuronal activity.

Changes in systemic physiological parameters such as blood pressure, respiratory and blood flow and oxygenation changes in extracerebral tissue layers can also affect fNIRS signal and may confound cerebral hemodynamics [137–140]. Skin blood flow is known to vary with a number of factors, including cardiac output, ambient temperature and, most strongly, sympathetic nervous system activity [141–145]. Such changes may, of course, be unrelated to the experimental procedure, but may also arise more systematically. For example, STN stimulation not only alleviates motor deficits but also influences autonomic regulation in patients with PD. Stemper et al. 2006 [146] revealed that during STN stimulation, the patients with PD showed an adequate cardiovascular response to orthostatic challenge with a stable blood pressure, a decrease in skin blood flow. While the systemic physiological interference distribute across all the channels over the head, our result showed relatively local spatial distribution of HbO and HbR concentration values mapped over the motor/premotor and frontal dorsolateral cortices (Fig 2B) using high-resolution optode distributed over most of the head (Fig 1B), however, we cannot completely exclude the influence of these confounding factors. Hence, one limitation of current study is that influence of DBS-induced decreases in skin blood flow cannot definitely be denied since changes in systemic physiological factors (e.g., cardiac, respiratory, and blood pressure fluctuations) can affect regional blood flow and some studies have revealed that skin blood flow changes, which can impact changes in HbO, are not homogeneous [147–149]. Therefore, the use of additional short-distance channels (<1 cm) is expected to provide a superior removal of the superficial systemic factors on functional near-infrared spectroscopy [149,150].

Lastly, we also took great care to minimize localization errors, particularly by using a dense array of optodes (32 paired sources and 16 detectors) and by digitizing their positions in each patient. Our previous work [128] with a more restricted arrangement in the premature infant made it possible to reconstruct (with adequate spatial resolution) the activation of the various perisylvian structures involved in the cortical coding of auditory stimuli. Although fNIRS (unlike fMRI) can only image the surface of the cortex, Eggebrecht et al. [151] showed that high-density fNIRS imaging is a practical, robust alternative to fMRI for mapping distributed cortical functions.

## Conclusions

The present fNIRS results provided new insights into the main cortical effects of STN-DBS -primarily an immediate reduction in blood flow in the primary SM area and the DLPFC. The cortical effects of STN-DBS neuromodulation are the same at rest and during performance of a task. Functional NIRS could be used to quantify cortical haemodynamics (especially in the frontal cortex) in individual patients, in order to screen for the potential side effects of STN-DBS and thus adjust stimulation parameters accordingly. In future studies, we propose a multimodal neuroimaging approach to combine EEG, fNIRS and high-density Diffuse Correlation Spectroscopy (DCS) to measure the effect of systemic blood flow in addition to cerebral hemodynamics. In addition, short distance channels setup for each source-detector pair would provide whole head information about systemic interferences.
